# Evolution of Protein-Mediated Biomineralization in Scleractinian Corals

**DOI:** 10.3389/fgene.2021.618517

**Published:** 2021-02-02

**Authors:** Tal Zaquin, Assaf Malik, Jeana L. Drake, Hollie M. Putnam, Tali Mass

**Affiliations:** ^1^Department of Marine Biology, The Leon H. Charney School of Marine Sciences, University of Haifa, Haifa, Israel; ^2^Department of Biological Sciences, University of Rhode Island, Kingston, RI, United States

**Keywords:** skeleton evolution, co-option, SOM proteins, stony corals, phylogenetic analysis

## Abstract

While recent strides have been made in understanding the biological process by which stony corals calcify, much remains to be revealed, including the ubiquity across taxa of specific biomolecules involved. Several proteins associated with this process have been identified through proteomic profiling of the skeletal organic matrix (SOM) extracted from three scleractinian species. However, the evolutionary history of this putative “biomineralization toolkit,” including the appearance of these proteins’ throughout metazoan evolution, remains to be resolved. Here we used a phylogenetic approach to examine the evolution of the known scleractinians’ SOM proteins across the Metazoa. Our analysis reveals an evolutionary process dominated by the co-option of genes that originated before the cnidarian diversification. Each one of the three species appears to express a unique set of the more ancient genes, representing the independent co-option of SOM proteins, as well as a substantial proportion of proteins that evolved independently. In addition, in some instances, the different species expressed multiple orthologous proteins sharing the same evolutionary history. Furthermore, the non-random clustering of multiple SOM proteins within scleractinian-specific branches suggests the conservation of protein function between distinct species for what we posit is part of the scleractinian “core biomineralization toolkit.” This “core set” contains proteins that are likely fundamental to the scleractinian biomineralization mechanism. From this analysis, we infer that the scleractinians’ ability to calcify was achieved primarily through multiple lineage-specific protein expansions, which resulted in a new functional role that was not present in the parent gene.

## Introduction

Scleractinian corals (commonly known as stony or hard corals) are foundation species in the tropical marine ecosystem (Moberg and Folke, 1999). One of their most important roles is reef formation through their ability to create a rigid aragonite exoskeleton by the process of biomineralization. These exoskeletons are valuable as they provide the ecological framework that supports high rates of primary production and permits extensive biological diversity in coral reef ecosystems (Veron et al., 2009), as well as serving as a large reservoir of biogenic calcium carbonate in the ocean (Cohen and McConnaughey, 2003). Scleractinians are among the oldest biomineralizing Metazoa, likely appearing in the late Ordovician (∼445 Million years ago), and becoming highly diverse (Stolarski et al., 2011; Drake et al., 2020). They are represented by different morphologies and spatial distribution (Veron, 2000), and show distinct molecular evolution, with the order split into two major clades, known as the Complexa (complex corals) and Robusta (robust corals) (Romano and Palumbi, 1996; Kitahara et al., 2010; Ying et al., 2018), named for the extent of skeletal calcification-specific patterns of corallite wall construction. While the process of biomineralization in scleractinians has long been studied (reviewed by Drake et al., 2020), its biomolecular mechanisms have only recently begun to be revealed (reviewed by Murdock, 2020), coinciding with advances in genomics and protein identification.

The “biomineralization toolkit” is the collective term for the many specific lipids, polysaccharides, and proteins both documented and hypothesized to be involved in the formation of the biomineral at various stages of an organism’s life history, some of which may become embedded in its skeleton (Livingston et al., 2006). Those organic molecules which are retained in the skeleton (the skeletal organic matrix, SOM) directly mediate and regulate the process by which many organisms from across all kingdoms of life form biominerals (Lowenstam and Weiner, 1989; Mann, 2001; Knoll, 2003), with the resulting biominerals exhibiting characteristics different from their abiogenic counterparts (Weiner, 2003; Gal et al., 2015). Out of all the SOM molecules, the most intensively studied are the proteins (Evans, 2019; Clark, 2020; Erwin, 2020; Murdock, 2020). Proteomic studies have shown that different lineages use sets of proteins with similar functional categories, including matrix formers, nucleation assisters, signalers, and remodelers to form their skeletal structure (Marin et al., 2016; Evans, 2019). Although the SOM proteins from distant organisms share common properties (Evans, 2019), each taxon-specific suite appears to have evolved independently through convergent and co-option evolution, resulting in varying contributions by lineage- and species-specific novel proteins, which exhibit contrasting rates of conservation between and within lineages (Drake et al., 2014).

In scleractinians, numerous SOM-related characteristics have been studied (Tambutté et al., 2011), yet only a few proteomic profiling experiments have been conducted, and then solely for tropical species. Extensive proteomic studies using species-specific genomes and transcriptomes include those of *Stylophora pistillata* (Drake et al., 2013; Peled et al., 2020), *Acropora millepora* (Ramos-Silva et al., 2013), and *Acropora digitifera* (Takeuchi et al., 2016), which, when combined, revealed over 100 SOM protein, hence members of the “biomineralization toolkit.” Similar to previous examinations of various metazoan lineages, scleractinian SOM proteins appear to share functional roles in carbohydrate-binding and catalytic activities (Ramos-Silva and Marin, 2015). Notably, the most extensively studied SOM proteins in scleractinians are the aspartic acid-rich proteins which assist in mineral nucleation and modification (Lowenstam and Weiner, 1989; Marin and Luquet, 2008; Mass et al., 2013; Gavriel et al., 2018; Laipnik et al., 2019), and α-carbonic anhydrases that play a role in both carbon supply and concentration (Bertucci et al., 2013; Zoccola et al., 2016). However, many scleractinian SOM proteins do not contain known functional domains and remain to be functionally characterized. Furthermore, out of all the known scleractinian SOM proteins, only a few were found to be shared between the three species (Takeuchi et al., 2016; Peled et al., 2020). The identification and characterization of the suite of scleractinian SOM proteins to date has led to the hypothesis that the proteins underlying scleractinian skeleton formation developed through stepwise evolution, supplementing proteins that are conserved across Metazoa with scleractinian-specific and species-specific novel proteins (Ramos-Silva et al., 2013; Takeuchi et al., 2016).

While useful for initial studies, most of the analyses that examined SOM protein diversity across taxa were carried out using heuristic methods of sequence similarity scores (e.g., BLAST), which estimates the phylogenetic relationships between a set of genes by the premise that higher-scoring sequence pairs are likely to have diverged more recently compared to their lower-scoring counterparts (Fitch, 1970; Lafond et al., 2018; Emms and Kelly, 2019). As a preliminary examination, sequence similarity can aid in determining homologous gene groups and are useful for function-related applications (Doyle et al., 2010; Paps and Holland, 2018; Richter et al., 2018); yet, the lack of a phylogenetic analysis based on a species tree limits our understanding of the proteins’ origin and evolutionary dynamics, as sequence duplication can result in a high sequence differentiation and subsequently leads to overlooking orthologous sequences (Lafond et al., 2018). To date, Bhattacharya et al. (2016) have published the most intensive phylogenetic study of the previously known scleractinian biomineralization proteins. The authors provided the basis for understanding scleractinian genomic evolutionary information, revealing mechanisms for scleractinians to adapt to changing environments while maintaining the ability to calcify. Recent advances in genome and transcriptome sequencing and the production of more gene databases are increasing our ability to provide a higher resolution comparison of SOM proteins and therefore, a better understanding of their evolutionary dynamics. This will allow extrapolation of the occurrence of the scleractinian “core biomineralization toolkit,” that is, the biomineralization-related proteins that are shared across scleractinian species and, as such, have a fundamental role in the skeleton formation process across the order. While at first glance, the most straightforward method may be to sequence more scleractinian skeletal proteomes, in practice, direct proteomic analyses are often incomplete and time-consuming (Marin et al., 2016; Aguilera et al., 2017; Peled et al., 2020); predictions based on transcriptomic and genomic data, therefore, become essential. However, on their own, such predictions can result in redundancies and overestimations, while at the same time overlooking potential gene candidates due to unresolved and incomplete genomes and transcriptomes (Eisenhaber, 2013; Sinha et al., 2018). Therefore, it is essential to combine both approaches, based on both proteins and DNA/RNA sequencing, to generate a more holistic picture of SOM protein evolution.

Here, we used a phylogenetic approach of the known scleractinian SOM proteins to study their evolution across the metazoan tree of life. As we have used the orthology/paralogy relationships for each protein in one species at a time, our results are independent for each lineage, providing a robust evaluation of their evolution. Our results reveal part of the “core biomineralization toolkit” across scleractinians, comprised of multiple proteins sharing an evolutionary history across distinct species. Since orthologous genes are more likely to share a biological function (Fang et al., 2010; Gabaldón and Koonin, 2013; Altenhoff et al., 2019), our approach might allow us to extrapolate the occurrence of proteins that play a fundamental role in the skeleton formation across scleractinians. However, the major fraction of each species SOM proteins were found to be independently co-opted into their own “biomineralization toolkit” from genes that evolved before the emergence of scleractinians. These were coupled with scleractinian-specific gene family expansions resulting in each scleractinian lineage and species having a unique set of SOM proteins.

## Materials and Methods

### Scleractinian SOM Protein Orthogroup and Gene Tree Reconciliation

**FIGURE 1:**
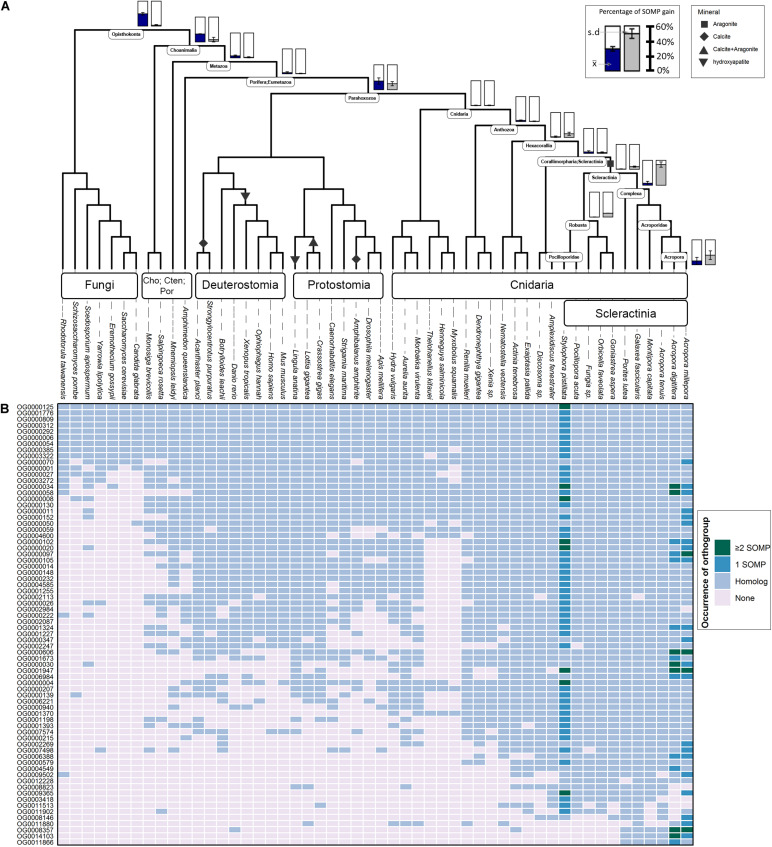
Evolutionary origin of SOM proteins (SOMP) and duplication rates across animal taxa. **(A)** In the species tree, each internal node represents an ancestral lineage, where tips represent extant species. Accordingly, blue-filled bars near specific internal nodes represent the percentage of known SOM proteins that were gained in a specific ancestral lineage, based on our results. Similarly, the gray-filled bar represents the percentage of known SOM proteins that evolved from a protein whose last duplication occurred at specific ancestral species. Bottom-right bars represent gain at extant species. The mean ($\bar{x}$) and standard deviation (s.d.) values are indicated in each bar graph. Black shapes symbolize the different mineral phases of the species that form a rigid skeleton or shell. **(B)** The heatmap describes the occurrences of each known SOM protein from *Stylophora pistillata*, *Acropora digitifera*, and *A. millepora* across the different orthogroups and their coding sequence homologs among all species in this study. For a comprehensive list of the SOM proteins found in each orthogroup, see Supplementary Table 2.

Our phylogenetic analysis was based on 43 annotated genomes spanning the metazoan kingdom (Supplementary Table 1), with the addition of seven Fungi species and two choanoflagellate species as the outgroups. The outgroups were selected in order to consider the Opisthokonta evolution. More specifically, the Fungi kingdom was included as scleractinians were found to share a complete histidine biosynthesis pathway, which is unique across the Metazoa (Ying et al., 2018), indicating that the consideration of fungi outgroup may be critical. Furthermore, the Choanoflagellata class was also included as it is considered to be the sister group of the Metazoa (King et al., 2008; Schalchian-Tabrizi et al., 2008). The complete dataset includes rigid skeleton/shell forming and non-forming taxa, including representation of major marine calcifying phyla (Mollusca, Echinodermata, Arthropoda, and Cnidaria) as we sought to group the known scleractinian SOM proteins (Drake et al., 2013; Ramos-Silva et al., 2013; Takeuchi et al., 2016; Peled et al., 2020) into their respective orthogroups. An orthogroup is defined as a set of genes descended from a single gene in the last common ancestor of all the species being considered. We limited our database of known scleractinian SOM proteins to studies for which the skeletal proteomes were sequenced against annotated genomes from the same species, limiting us to representatives of scleractinian SOM proteins from *A. digitifera, A. millepora*, and *S. pistillata*. We decided to use a larger proportion of species from the cnidarian phylum and more particularly, within the scleractinian order, as they are the focus of this study. The annotated genomes in the analysis are comprised of datasets with a median BUSCO score of 90.05% (Seppey et al., 2019; Supplementary Table 1). To infer the scleractinian SOM orthogroups, we clustered all the protein-coding sequences from our entire database using OrthoFinder 2.2.7 (Emms and Kelly, 2015, 2019), to give a total of ∼16,000 orthogroups. After identifying all orthogroups from each sequence in our known SOM protein dataset, we aligned the sequences in each orthogroup separately using MAFFT (Katoh and Standley, 2013), followed by the removal of sequences and regions based on inconsistencies in the consensus alignment; sequences with both fewer than thirty aligned amino acids and less than 50% of the sequence aligned columns with <2 aligned sequences were removed. Gene trees were constructed in IQ-TREE (Nguyen et al., 2015) using the best-fitted model (LG) and discrete Gamma distribution of four rates across site categories (Resulting trees can be found in Supplementary File 1). To infer pairwise orthology relationships and to reconstruct the sequences’ evolutionary histories, the gene trees were further rooted and reconciled via the Orthofinder2 pipeline, using the rooted species tree with the topology presented in Figure 1A, which is based on the current knowledge of animal phylogeny (Laumer et al., 2019; Fernández and Gabaldón, 2020). Then we selected orthogroups that include known SOM proteins of *A. digitifera, A. millepora*, and *S. pistillata*. Lastly, the known scleractinian SOM protein orthogroups were used for downstream analyses.

As data acquired from *de novo* transcriptomes are highly fragmented and can lead to misinterpretation of downstream analysis (Emms and Kelly, 2015), we did not include any transcriptomic data sets as part of the Orthofinder2 pipeline. However, the ability to produce a calcium carbonate rigid skeleton is not unique to the scleractinian order within Cnidaria, with several taxa, including hydrozoans and octocorals, demonstrating this ability. Therefore, to further identify common molecular traits between extant taxa, we searched for the known scleractinian SOM proteins putative orthologs in the transcriptome of a representative species, the blue octocoral *Heliopora coerulea* (Guzman et al., 2018) using Conditional Reciprocal Best BLAST 0.6.6 (CRB-BLAST) (Aubry et al., 2014), that performs complementary BLAST alignments between query and target sequences. Sequences with more than a single hit across query and target of the same scleractinian species, were removed.

### Scleractinian SOM Protein Orthogroup Gain and Duplication Patterns

We based our analysis on Dollo’s parsimony (Farris, 1977) and phylostratigraphic profiling (Domazet-Lošo et al., 2007) to infer the likely phylogenetic origin of each known scleractinian SOM gene family. Dollo’s parsimony, implemented in COUNT 9.1106 (Csûös, 2010), is modeled on Dollo’s law (Dollo, 1893) that argues the statistical improbability of an organism to transition into a different state. It leads to a substantial simplification of evolutionary scenarios as it assumes that genes, which have been lost during evolution in a particular lineage, are unlikely to be regained. This heuristic approach enables us to map the scleractinian SOM proteins to the species tree based on the most phylogenetically distant lineage present in their representative orthogroup and to determine if SOM protein gains are the product of a lineage-specific evolution rather than the co-option of pre-existing genes into a skeleton formation role. If an SOM protein was reported as not having an orthogroup, we assigned it as a species-specific protein.

Gene duplication rates were calculated using the phylogenetic birth-and-death model implemented in COUNT 9.1106 (Csûös, 2010). Specifically, the rate model was calculated and optimized under the gain-loss–duplication model with the Poisson distribution at the root. The variation rate across families was set to 4:1:1:4 gamma categories for the edge length, the loss rate, gain rate, and the duplication rate, respectively. The convergence criteria applied were set to 100 rounds for the optimization rounds with a likelihood threshold of 0.1. This model is based on the assumption that the primary mechanism of gene gain in eukaryotes is genomic duplications, while the possibility of gene gain through horizontal gene transfer (HGT) between different nodes of the gene tree is less likely (Csûrös and Miklós, 2006). However, as our methodological approach centered around using gene phylogenies to infer pairwise orthology and paralogy relationships for all genes in the analysis, it is robust to the effect of HGT, genome completeness and variable genome size (Huerta-Cepas et al., 2014; Emms and Kelly, 2015, 2019).

### SOM-Enriched Branches and Permutation Test Method

In each rooted gene tree (see above), we first detected “Scleractinia branches,” namely: groups of proteins that evolved from a single protein that existed in the last common ancestor of complex/robust scleractinians. For each scleractinian branch, we calculated the following values: (1) the total number of proteins in that branch, PN; (2) the number of SOM proteins known from previous studies (Drake et al., 2013; Ramos-Silva et al., 2013; Takeuchi et al., 2016; Peled et al., 2020) in that branch, pn; and (3) SOM protein density, pn/PN, if at least one known SOM protein was observed, otherwise density = 0. Our analysis tested whether known scleractinian SOM proteins evolved independently as opposed to having evolved from an SOM-related protein that existed in the most recent common ancestor of scleractinia. We compared our observed “Scleractinia branches” SOM protein density to an expected density obtained by randomly selecting (n1) *S. pistillata*, (n2) *A. digitifera*, and (n3) *A. millepora* proteins (where n1, 2, 3 are equal to the counts of respective observed SOM proteins). The comparison was conducted using a permutation test (*n* = 1,000 sums), where a *P*-value was defined as the proportion of cases where the observed sum of density is smaller than the expected sum. We also repeated this test where density = 1 is assigned if species from both the complex and robust scleractinian clade are found in a cluster, and otherwise density = 0.

## Results

### Scleractinian SOM Protein Evolutionary History

Using OrthoFinder2, 123 known scleractinian SOM proteins were clustered in 72 different orthogroups (i.e., all genes descended from a single gene belonging to the last common ancestor of the tested species). The identification of known scleractinian SOM proteins was based on previously published scleractinian SOM proteomes (Figure 1B and Supplementary Table 2). The majority of the orthogroups, 54 out of 72, were found to include known SOM proteins from a single species, while twelve orthogroups contain two species and seven orthogroups are represented by all three species (Figure 1B and Supplementary Table 1). In fourteen orthogroups, we have identified the occurrence of scleractinian known SOM protein paralogs (that is, proteins which separated by a duplication event), from one or more species (Figure 1B and Supplementary Table 2). Although most orthogroups included known SOM proteins from a single species, we identified orthologs of those proteins across most scleractinian species with a mean of 67.5 (±1.78) orthogroups per scleractinian species (Figure 1B).

Gene gain can involve the co-option of pre-existing molecular traits to serve a new functional role as well as the evolution of lineage-specific genes, through a multiple “birth” model (True and Carroll, 2002; Choi and Kim, 2006; McLennan, 2008; Mello et al., 2018). As such, we evaluated these two categories using a phylostratigraphic approach. We observed an unbalanced gene distribution, where 76% of the SOM proteins are descendants of genes that were gained before the cnidarian diversification (Figure 1A and Supplementary Table 3). In our analysis, we found only a single scleractinian-specific orthogroup (OG0012228), representing species from both the robust and complex scleractinian clades. This orthogroup contains the *S. pistillata* SOM protein “Coral Acid-Rich Protein 2” (Supplementary Figure 1), which was observed to have an essential role in the early life stages of scleractinians (Mass et al., 2016; Akiva et al., 2018). However, ∼60% of the SOM proteins, found in the different orthogroups, emerged during the scleractinian evolution due to gene family expansion (Figure 1A and Supplementary Table 3). Simultaneously, 10.53 and 7.41% of the known SOM proteins from *S. pistillata* and *A. millepora*, respectively, were not found in any a specific orthogroup (Supplementary Table 2), suggesting a species-specific evolution.

Calculation of duplication rates across the tested orthogroups (Supplementary Table 3) shows high rates at the Opisthokonta and Choanimalia branches, followed by low rates at the subsequent branches leading to the known SOM proteins’ species (Supplementary Figure 2).

Using CRB-BLAST, 21 distinct transcripts from the massive aragonite skeleton forming octocoral, *H. coerulea*, were identified to be putative orthologs of 27 scleractinian known biomineralization proteins, spanning 20 orthogroups (Supplementary Table 4). All identified *H. coerulea* transcripts were present in orthogroups with the phylogenetic origin at the metazoan branch or earlier (Figure 1B). Furthermore, in all of the respective orthogroups, at least two other non-rigid skeleton forming octocoral species were identified (Figure 1B). In five instances, the *H. coerulea* transcripts were identified as putative orthologs of more than a single species in the same orthogroup. Those orthogroups include putative enzymes, transporters, and acidic proteins, suggesting functional conservation across lineages. However, due to limitations of the transcriptomic dataset, the evolutionary relationship between sequences was not resolved. Our understanding of the evolutionary dynamics that guide the biomineralization gene repertoire evolution will increase through the growth in annotated genomic datasets.

### Identification of Species-Specific vs. Scleractinian-Conserved SOM Protein Evolution

**FIGURE 2:**
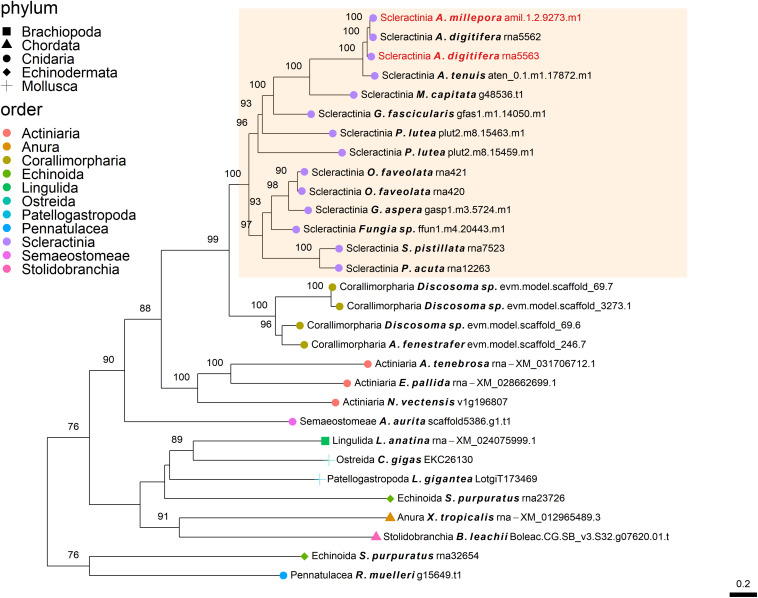
Rooted gene tree of polycystin-like proteins (OG0006984) representing the independent co-option of orthologous proteins into the SOM of the Acroporidae species. Node points represent the phylum (shape) and order (color). Tips that are labeled red indicate the occurrence of scleractinian known SOM proteins. The highlighted cluster represents a “Scleractinia branch.” Node support values indicate percentage bootstrap values. Only values above 75 are indicated.

**FIGURE 3:**
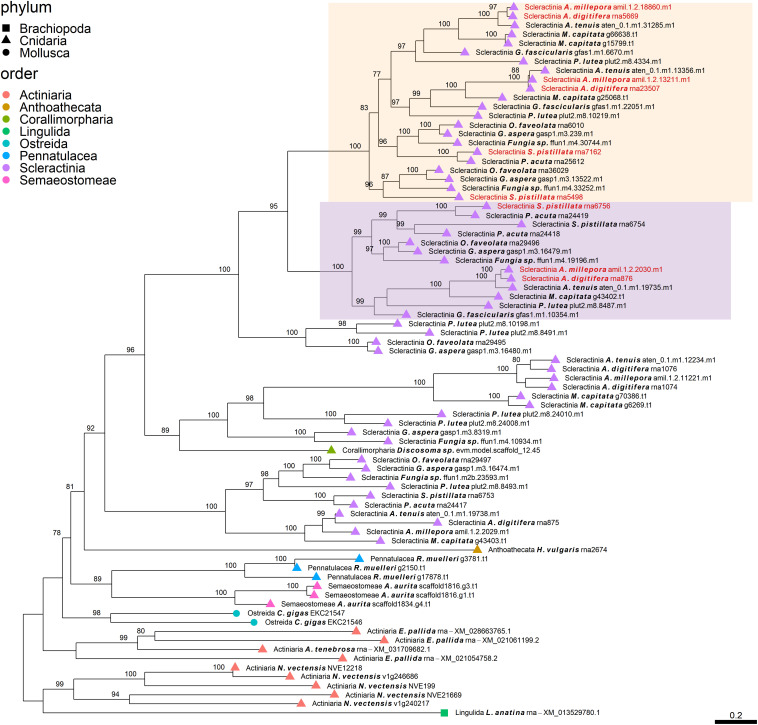
Rooted gene tree of scleractinian coral aspartic acid-rich proteins (OG0001947) representing the scleractinian-specific expansion of the gene family involving species from both the complex and robust scleractinian clades. Node points represent the phylum (shape) and order (color). Tips that are labeled red indicate the occurrence of scleractinian known SOM proteins. The two highlighted clades represent orthologous relationships between scleractinian sequences to the known SOM protein(s) in their respective “Scleractinia branch.” Node support values indicate percentage bootstrap values. Only values above 75 are indicated.

**FIGURE 4:**
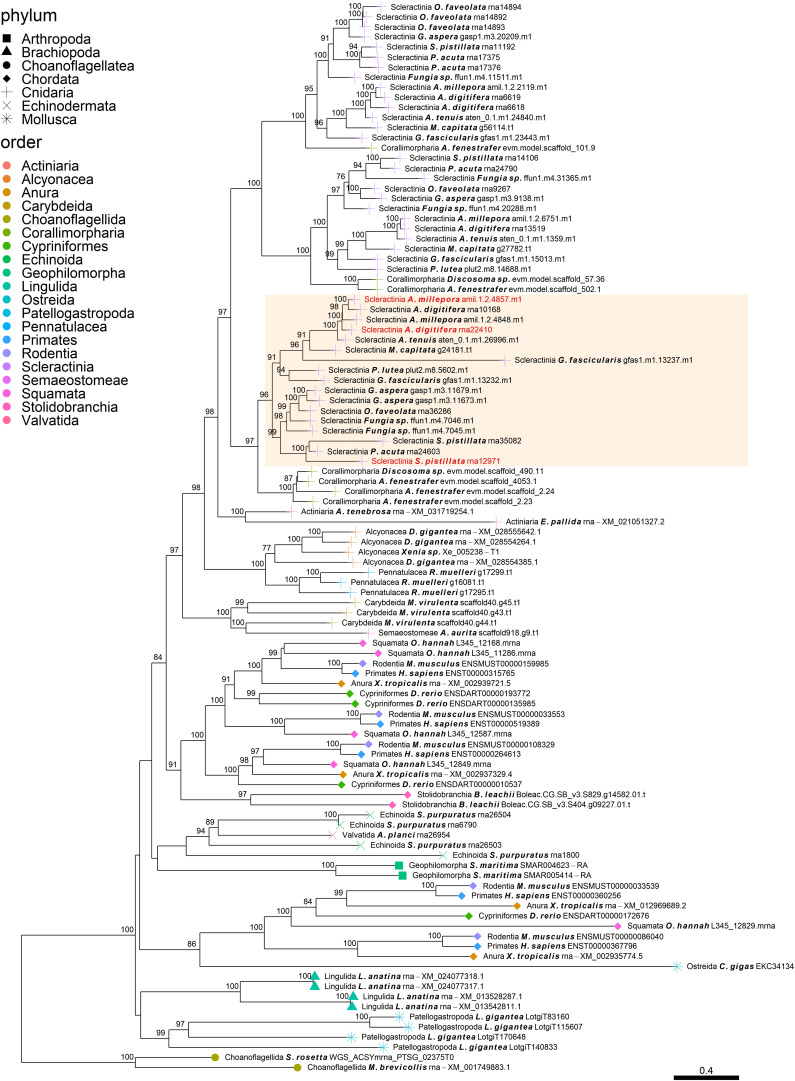
Rooted gene tree of metal transport proteins (OG0001324) representing the scleractinian-specific expansion of the gene family. Node points represent the phylum (shape) and order (color). Tips that are labeled red indicate the occurrence of scleractinian known SOM proteins. The highlighted cluster represents a “Scleractinia branch.” Node support values indicate percentage bootstrap values. Only values above 75 are indicated.

In the 72 gene trees tested, we detected multiple “Scleractinia branches,” namely: groups of scleractinian genes that evolved from a single gene copy of the most recent common ancestor of scleractinians. Subsequently, we identified known SOM proteins in *S. pistillata* (Drake et al., 2013; Peled et al., 2020), *A. digitifera* (Takeuchi et al., 2016), and *A. millepora* (Ramos-Silva et al., 2013) within each “Scleractinia branch.” Accordingly, in 12 “Scleractinia branches,” we observed the cross-species conservation of more than a single known SOM protein (Figure 2 and Table 1). Moreover, in eight “Scleractinia branches,” known SOM proteins from both the complex and robust scleractinian clades were identified (Figures 3, 4 and Table 1). A permutation test revealed that this clustering pattern is non-random (*p* < 0.001), with a significantly higher SOM protein density per cluster than expected (Table 1 and Supplementary Figure 2). Overall, these results indicate that the evolution of SOM-related functions emerged in the last common ancestor of scleractinians.

**TABLE 1:** Known coral SOM proteins in “Scleractinia branches.”

**Orthogroup**	**Orthogroup description**	**SOM protein occurrences in “Scleractinia branch”**	**Size of “Scleractinia branch”**	**SOM protein density in “Scleractinian branch”**
OG0000020	Trypsin	2	4	0.5
OG0000030	Uncharacterized SOM protein 5	3	40	0.075
OG0000034*	Properdin	3	11	0.273
OG0000058*	Pikachurin-like	5	33	0.152
OG0000097*	MAM and LDL-receptor domain-containing proteins	3	11	0.273
OG0000102*	Protocadherin	2	16	0.125
OG0000105*	ZP domain-containing proteins	3	16	0.186
OG000606*	Galaxin-2	5	18	0.278
OG0001324*	Hephestin	3	18	0.167
OG0001947*	Aspartic acid-rich proteins	6	23	0.261
OG0006984	Polycystin-like proteins	2	22	0.091
OG0009365	Uncharacterized SOM protein 8	2	11	0.182

*The list represents only “Scleractinia branches” with at least two scleractinian known SOM proteins and their density in the branch. Asterisks symbolize “Scleractinia branches” that contain SOM proteins from species of both the complex and robust scleractinian clades.*

## Discussion

The mineralized skeleton is a paramount innovation, appearing simultaneously across phyla during the Cambrian Explosion (Murdock and Donoghue, 2011; Erwin, 2020; Murdock, 2020). Currently, there is growing evidence that many animal taxa inherited sets of ancestral genes that were then independently co-opted to guide skeleton formation (Murdock, 2020). Here, we sought to examine the evolutionary history of scleractinian biomineralization proteins across the metazoan tree of life to determine how modern stony corals evolved to form one of the most significant biostructures on Earth (Veron et al., 2009).

Determining the age of the SOM proteins by phylostratigraphy (Figure 1A) indicates that each scleractinian species’ “biomineralization toolkit” has a similar age profile, characterized by progressively descending gene gain toward the tip of the tree. The most substantial proportion of genes for which orthogroups were found, appear to have evolved over 700 million years ago, before the cnidarian diversification, through extensive expansions of gene families. Of these ancestral genes, 96% of orthogroups contain at least one species that does not form a rigid skeleton (Figure 1B). The evidence that the scleractinian SOM proteins have an ancient origin and are shared between rigid skeleton forming and non-forming taxa further supports the hypothesis that the “biomineralization toolkit” evolved by the differential independent co-option of genes that had unrelated skeleton forming functions (Erwin, 2020).

**FIGURE 5:**
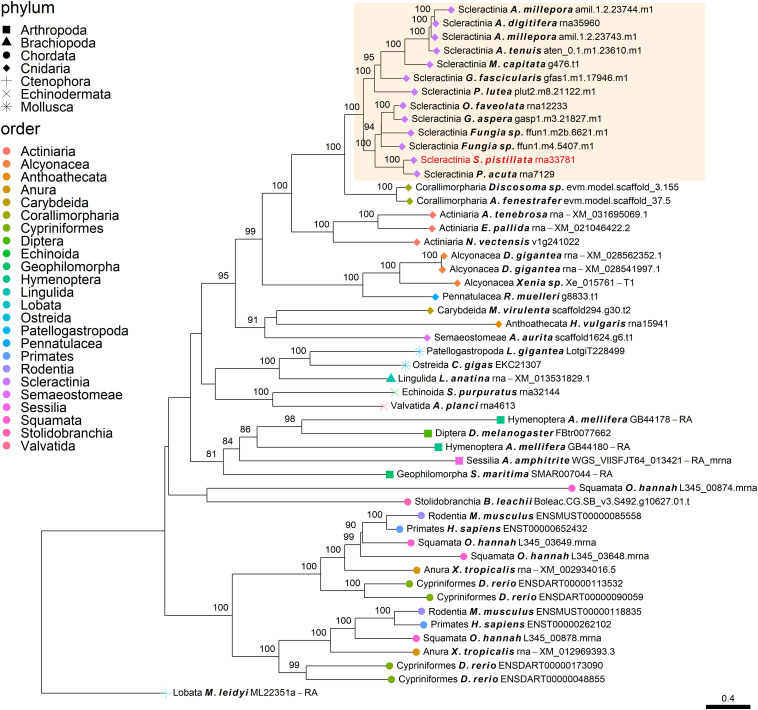
Rooted gene tree of CAP-Gly domain-containing proteins (OG0004585) representing the independent co-option of a single *S. pistillata* protein into the SOM. Node points represent the phylum (shape) and order (color). Tips that are labeled red indicate the occurrence of known SOM proteins. The highlighted cluster represents a “Scleractinia branch.” Node support values indicate percentage bootstrap values. Only values above 75 are indicated.

The independent co-option of ancient genes is not only restricted to between-lineage relationships, but is also evident in the same lineage, with taxa utilizing a unique set of proteins with similar functional patterns (Evans, 2019). For example, mollusks have been found to express in their mantle a species-specific unique set of genes that evolved before their evolutionary origins (Aguilera et al., 2017). This tendency for different species to use a separate set of ancient genes that converge toward the same results is also found in stony corals. Orthogroup OG0006984 (Figure 2), was found to include polycystin-like sequences, having calcium binding sites (Rastogi and Liberles, 2005). This gene family has an evolutionary origin going back at least to the Parahoxozoa lineage. Although the SOM proteins were found to be orthologous to sequences from all three scleractinian species with a published SOM proteome, only the Acroporidae family proteins has been identified in the skeleton. In addition, solely an *S. pistillata* known SOM protein was identified in the CAP-Gly orthogroup (OG0004585, Figure 5) which contain genes that are involved in the transport of vesicles along the cytoskeletal network (Riehemann and Sorg, 1993). Similarly to the polycystin orthogroup, the known SOM protein shares a 1:1 orthology to sequences from the scleractinian species with a published SOM proteome yet, was only identified in the *S. pistillata*’s skeleton. It is noteworthy that this example can be a result of the identification of different protein sets by the use of diverse protein extraction methods, which can lead not only to different yield but also different content of proteins as the various methods are biased toward their own properties (Marin et al., 2016; Klont et al., 2018; Peled et al., 2020). For example, mechanical filtration is biased toward hydrophobic proteins, while acetone precipitation increase the identification of hydrophilic proteins (Thongboonkerd et al., 2002). As such, the use of multiple extraction methods to retrieve SOM proteins makes it difficult to fully compare across species, likely leading to underestimation of the SOM protein repertoire.

Species-specific novel proteins are those with no orthologous relationship outside the species of interest. This class of proteins was also found to contribute to the scleractinian SOM protein assemblage, although to a lesser extent than the co-option of pre-existing molecular traits (Figure 1A and Supplementary Table 2). While the variability in their detection is high between our species of interest, as discussed above, it could also be caused by underestimation due to different extraction methods. In mollusks, species-specific proteins have been suggested to play a considerable role in physiological adaptations to environmental changes (Arivalagan et al., 2016) and in the formation of the numerous shell morphologies and properties (Kocot et al., 2016). However, to date, our knowledge of the functional roles of most SOM proteins in scleractinians is still lacking. Therefore, the clarification of novel biomineralization proteins from different species, combined with experimental functional validation, is still required to elucidate their significance.

Gene duplication events play a crucial role in the emergence of novel genes (Singh and Bansal, 2019), and are thought to have contributed to the evolution of morphological and physiological diversity (True and Carroll, 2002; Kondrashov, 2012; Lallemand et al., 2020). The high rates of duplications that occurred before the metazoan diversification (Supplementary Figure 2) suggest that both ancient duplications and the retention of duplicated genes have contributed to the expansion of the gene families. This allowed the emergence of novel functions and possibly promoting the specific evolution of scleractinian SOM proteins. The mechanisms that may lead to gene duplications in scleractinian lineages include tandem duplications, transposable elements, retrotransposition and transduplication, and segmental duplications (Lallemand et al., 2020). Duplicated genes can acquire novel functions, namely, undergoing neofunctionalization and subfunctionalization, where paralogs may carry complementary functions (Thongboonkerd et al., 2002). As we further explain here, it seems that the scleractinian SOM protein evolution is characterized by the gain of SOM-specific genes, supporting a neofunctionalization model.

Despite the many scleractinian SOM protein gene families that appear to be orthologous to diverse phyla, the evolution of scleractinian-specific SOM lineages and their neofunctionalization seem to be the primary force (Figure 1A). For example, the aspartic acid-rich gene family (Figure 3) is represented by several known SOM proteins from all three scleractinian species with sequenced skeletal proteomes. While known coral SOM proteins in this orthogroup have several orthologous sequences across the cnidarian, molluscan and brachiopod phyla, the orthogroup expansion resulted in the speciation of two distinct scleractinian-specific clusters, each with its own unique last common ancestor (i.e., being spread across the robust and complex clades). A similar example can be found for the metal transport gene tree family (Figure 4), where the various scleractinian SOM proteins share diverse orthologous relationships across many phyla, nonetheless converging into a scleractinian-specific branch. Although the scleractinian “biomineralization toolkit” bound in the skeleton seems to differ between species, the presence of multiple orthologs to known SOM proteins seems to indicate that SOM-related functionality emerged in the last common ancestor of scleractinia (Table 1). As such, these known SOM orthologs retained a fundamental role in biomineralization and are therefore conserved across scleractinian species.

Going further, we propose that for distinct scleractinian species whose skeletal proteomes have not yet been sequenced, the likelihood of orthologous genes to known SOM proteins found under specific “Scleractinia branches,” will be further identified upon proteomic profiling. Subsequently, the ability to have SOM-related gene markers will increase our capability to predict corals’ response to changing environments, without the need to perform proteomic analysis on a large number of coral species. However, we acknowledge that our predictions should be taken with certain caveats. While proteomic data representing both the complex and robust scleractinian clades are available, our resolution may be limited, as very few scleractinian species possess a published SOM proteome profile relative to the ∼1,600 known scleractinian species (WoRMS, 2020). Consequently, we are likely missing groups of SOM proteins conserved across species and affiliated with specific growth forms, life strategies and habitats. As such, we suggest that the type of analysis used here will become more robust with the addition of a diverse representation of scleractinian SOM proteomes that will further help identify the “core biomineralization toolkit” across, and between, the scleractinian order.

Altogether, our results clarify the differing evolutionary dynamics of the scleractinian corals’ “biomineralization toolkit” as illustrated in Supplementary Figure 4. First, we provide further evidence that the evolution of a subset of biomineralization proteins in scleractinians is through a stepwise process (Ramos-Silva et al., 2013; Takeuchi et al., 2016; Murdock, 2020). This is evident by the presence of pre-existing genes shared by an assortment of skeleton forming and non-forming taxa. It suggests that gene co-option played an integral role in the initial development of an extracellular organic matrix in the last common ancestor of the scleractinian order. Second, the differential independent co-option, through gene duplications, followed by sub and neofunctionalization to form lineage-specific proteins and construct species-specific organic matrix frameworks, can have a significant role in distinct skeleton morphology between species. Third, this would supplement the contribution of novel species-specific proteins, crucially allowing organismal plasticity and adaptation to environmental change. While the presence of such lineage- and species-specific key innovations appears to have crucial roles, our results emphasize the importance of the evolutionary dynamics through gene duplications, although the mechanism remains to be revealed.

## Data Availability Statement

The datasets presented in this study can be found in online repositories. The names of the repository/repositories and accession number(s) can be found below: https://github.com/OOassafOO/gene-set-overrepresentation-in-trees.

## Author Contributions

TZ, AM, and TM designed the study. TZ and AM analyzed the data. All authors wrote the manuscript and approved it.

## Conflict of Interest

The authors declare that the research was conducted in the absence of any commercial or financial relationships that could be construed as a potential conflict of interest.

## References

[B1] Aguilera F. McDougall C. Degnan B. M. Irwin D. (2017). Co-option and de novo gene evolution underlie molluscan shell diversity. *Mol. Biol. Evol.* 34 779–792. 10.1093/molbev/msw294 28053006 PMC5400390

[B2] Akiva A. Neder M. Kahil K. Gavriel R. Pinkas I. Goobes G. (2018). Minerals in the pre-settled coral *Stylophora pistillata* crystallize via protein and ion changes. *Nat. Commun.* 9:1880. 10.1038/s41467-018-04285-7 29760444 PMC5951882

[B3] Altenhoff A. M. Glover N. M. Dessimoz C. (2019). *Inferring orthology and paralogy in Methods in Molecular Biology.* Totowa: Humana Press Inc 149–175. 10.1007/978-1-4939-9074-0_5 31278664

[B4] Arivalagan J. Yarra T. Marie B. Sleight V. A. Duvernois-Berthet E. Clark M. S. (2016). Insights from the shell proteome: biomineralization to adaptation. *Mol. Biol. Evol.* 34 66–77. 10.1093/molbev/msw219 27744410 PMC5854119

[B5] Aubry S. Kelly S. Kümpers B. M. C. Smith-Unna R. D. Hibberd J. M. (2014). Deep evolutionary comparison of gene expression identifies parallel recruitment of trans-factors in two independent origins of C_4_ photosynthesis. *PLoS Genet.* 10:1004365. 10.1371/journal.pgen.1004365 24901697 PMC4046924

[B6] Bertucci A. Moya A. Tambutté S. Allemand D. Supuran C. T. Zoccola D. (2013). Carbonic anhydrases in anthozoan corals-A review. *Bioorg. Med. Chem.* 21 1437–1450. 10.1016/j.bmc.2012.10.024 23199478

[B7] Bhattacharya D. Agrawal S. Aranda M. Baumgarten S. Belcaid M. Drake J. L. (2016). Comparative genomics explains the evolutionary success of reef-forming corals. *Elife* 5 1–26. 10.7554/eLife.13288 27218454 PMC4878875

[B8] Choi I. G. Kim S. H. (2006). Evolution of protein structural classes and protein sequence families. *Proc. Natl. Acad. Sci. U. S. A.* 103 14056–14061. 10.1073/pnas.0606239103 16959887 PMC1560931

[B9] Clark M. S. (2020). Molecular mechanisms of biomineralization in marine invertebrates. *J. Exp. Biol.* 223:jeb206961. 10.1242/jeb.206961 32471885 PMC7272335

[B10] Cohen A. L. McConnaughey T. A. (2003). Geochemical perspectives on coral mineralization. *Rev. Mineral. Geochem.* 54 151–187. 10.2113/0540151 28159795

[B11] Csûös M. (2010). Count: evolutionary analysis of phylogenetic profiles with parsimony and likelihood. *Bioinformatics* 26 1910–1912. 10.1093/bioinformatics/btq315 20551134

[B12] Csûrös M. Miklós I. (2006). “A probabilistic model for gene content evolution with duplication, loss, and horizontal transfer,” in *Research in Computational Molecular Biology*, eds Apostolico A. Guerra C. Istrail S. Pevzner P. A. Waterman M. (Heidelberg: Springer Berlin Heidelberg), 206–220.

[B13] Dollo L. (1893). The laws of evolution. *Bull. Soc. Bel. Geol. Paleontol.* 7 164–166.

[B14] Domazet-Lošo T. Brajković J. Tautz D. (2007). A phylostratigraphy approach to uncover the genomic history of major adaptations in metazoan lineages. *Trends Genet.* 23 533–539. 18029048 10.1016/j.tig.2007.08.014

[B15] Doyle M. A. Gasser R. B. Woodcroft B. J. Hall R. S. Ralph S. A. (2010). Drug target prediction and prioritization: using orthology to predict essentiality in parasite genomes. *BMC Genomics* 11:1–14. 20361874 10.1186/1471-2164-11-222 PMC2867826

[B16] Drake J. L. Mass T. Falkowski P. G. (2014). The evolution and future of carbonate precipitation in marine invertebrates: witnessing extinction or documenting resilience in the Anthropocene? *Elem. Sci. Anthr.* 2:000026 10.12952/journal.elementa.000026

[B17] Drake J. L. Mass T. Haramaty L. Zelzion E. Bhattacharya D. Falkowski P. G. (2013). Proteomic analysis of skeletal organic matrix from the stony coral *Stylophora pistillata*. *Proc. Natl. Acad. Sci.* 110 3788–3793. 10.1073/pnas.1301419110 23431140 PMC3593878

[B18] Drake J. L. Mass T. Stolarski J. Von Euw S. Schootbrugge B. Falkowski P. G. (2020). How corals made rocks through the ages. *Glob. Chang. Biol.* 26 31–53. 10.1111/gcb.14912 31696576 PMC6942544

[B19] Eisenhaber F. (2013). *“Prediction of protein function,” in Discovering Biomolecular Mechanisms with Computational Biology.* Boston, MA: Springer US, 39–54. 10.1007/0-387-36747-0_4

[B20] Emms D. M. Kelly S. (2015). OrthoFinder: solving fundamental biases in whole genome comparisons dramatically improves orthogroup inference accuracy. *Genome Biol.* 16:157. 10.1186/s13059-015-0721-2 26243257 PMC4531804

[B21] Emms D. M. Kelly S. (2019). OrthoFinder: phylogenetic orthology inference for comparative genomics. *Genome Biol.* 20:238. 10.1186/s13059-019-1832-y 31727128 PMC6857279

[B22] Erwin D. H. (2020). The origin of animal body plans: a view from fossil evidence and the regulatory genome. *Development* 147:dev182899. 10.1242/dev.182899 32079678

[B23] Evans J. S. (2019). The biomineralization proteome: protein complexity for a complex bioceramic assembly process. *Proteomics* 2019:1900036. 10.1002/pmic.201900036 31219243

[B24] Fang G. Bhardwaj N. Robilotto R. Gerstein M. B. (2010). Getting started in gene orthology and functional analysis. *PLoS Comput. Biol.* 6:e1000703–e1000703. 10.1371/journal.pcbi.1000703 20361041 PMC2845645

[B25] Farris J. S. (1977). Phylogenetic analysis under Dollo’s law. *Syst. Biol.* 26 77–88. 10.1093/sysbio/26.1.77

[B26] Fernández R. Gabaldón T. (2020). Gene gain and loss across the metazoan tree of life. *Nat. Ecol. Evol.* 4:1069–x. 10.1038/s41559-019-1069-x 31988444 PMC7124887

[B27] Fitch W. M. (1970). Distinguishing homologous from analogous proteins. *Syst. Zool.* 19 99–113. 5449325

[B28] Gabaldón T. Koonin E. V. (2013). Functional and evolutionary implications of gene orthology. *Nat. Rev. Genet.* 14 360–366. 10.1038/nrg3456 23552219 PMC5877793

[B29] Gal A. Weiner S. Addadi L. (2015). A perspective on underlying crystal growth mechanisms in biomineralization: solution mediated growth versus nanosphere particle accretion. *Cryst. Eng. Comm* 17 2606–2615. 10.1039/c4ce01474j

[B30] Gavriel R. Nadav-Tsubery M. Glick Y. Yarmolenko A. Kofman R. Keinan-Adamsky K. (2018). The coral protein CARP3 acts from a disordered mineral surface film to divert aragonite crystallization in favor of Mg-calcite. *Adv. Funct. Mater.* 28:1707321 10.1002/adfm.201707321

[B31] Guzman C. Shinzato C. Lu T. M. Conaco C. (2018). Transcriptome analysis of the reef-building octocoral, *Heliopora coerulea*. *Sci. Rep.* 8 1–11. 10.1038/s41598-018-26718-5 29849113 PMC5976621

[B32] Huerta-Cepas J. Capella-Gutiérrez S. Pryszcz L. P. Marcet-Houben M. Gabaldón T. (2014). PhylomeDB v4: Zooming into the plurality of evolutionary histories of a genome. *Nucleic Acids Res.* 42 D897–D902. 10.1093/nar/gkt1177 24275491 PMC3964985

[B33] Katoh K. Standley D. M. (2013). MAFFT multiple sequence alignment software version 7: improvements in performance and usability. *Mol. Biol. Evol.* 30 772–780. 10.1093/molbev/mst010 23329690 PMC3603318

[B34] King N. Westbrook M. J. Young S. L. Kuo A. Abedin M. Chapman J. (2008). The genome of the choanoflagellate Monosiga brevicollis and the origin of metazoans. *Nature* 451 783–788. 10.1038/nature06617 18273011 PMC2562698

[B35] Kitahara M. V. Cairns S. D. Stolarski J. Blair D. Miller D. J. (2010). A comprehensive phylogenetic analysis of the Scleractinia (Cnidaria, Anthozoa) based on mitochondrial CO1 sequence data. *PLoS One* 5:e11490. 10.1371/journal.pone.0011490 20628613 PMC2900217

[B36] Klont F. Bras L. Wolters J. C. Ongay S. Bischoff R. Halmos G. B. (2018). Assessment of sample preparation bias in mass spectrometry-based proteomics. *Anal. Chem.* 90 5405–5413. 10.1021/acs.analchem.8b00600 29608294 PMC5906755

[B37] Knoll A. H. (2003). Biomineralization and evolutionary history. *Rev. Mineral. Geochem.* 54 329–356. 10.2113/0540329 28159795

[B38] Kocot K. M. Aguilera F. McDougall C. Jackson D. J. Degnan B. M. (2016). Sea shell diversity and rapidly evolving secretomes: insights into the evolution of biomineralization. *Front. Zool.* 13:23. 10.1186/s12983-016-0155-z 27279892 PMC4897951

[B39] Kondrashov F. A. (2012). Gene duplication as a mechanism of genomic adaptation to a changing environment. *Proc. R. Soc. B Biol. Sci.* 279 5048–5057. 10.1098/rspb.2012.1108 22977152 PMC3497230

[B40] Lafond M. Meghdari Miardan M. Sankoff D. (2018). Accurate prediction of orthologs in the presence of divergence after duplication. *Bioinformatics* 34 i366–i375. 29950018 10.1093/bioinformatics/bty242 PMC6022570

[B41] Laipnik R. Bissi V. Sun C. Y. Falini G. Gilbert P. U. P. A. Mass T. (2019). Coral acid rich protein selects vaterite polymorph in vitro. *J. Struct. Biol.* 209 107431. 10.1016/j.jsb.2019.107431 31811894 PMC7058422

[B42] Lallemand T. Leduc M. Landčs C. Rizzon C. Lerat E. (2020). An overview of duplicated gene detection methods: Why the duplication mechanism has to be accounted for in their choice. *Genes* 11:1046. 10.3390/genes11091046 32899740 PMC7565063

[B43] Laumer C. E. Fernández R. Lemer S. Combosch D. Kocot K. M. Riesgo A. (2019). Revisiting metazoan phylogeny with genomic sampling of all phyla. *Proc. R. Soc. B Biol. Sci.* 286:20190831. 10.1098/rspb.2019.0831 31288696 PMC6650721

[B44] Livingston B. T. Killian C. E. Wilt F. Cameron A. Landrum M. J. Ermolaeva O. (2006). A genome-wide analysis of biomineralization-related proteins in the sea urchin *Strongylocentrotus purpuratus*. *Dev. Biol.* 300 335–348. 10.1016/j.ydbio.2006.07.047 16987510

[B45] Lowenstam H. A. Weiner S. (1989). *On biomineralization.* Oxford: Oxford University Press on Demand.

[B46] Mann S. (2001). *Biomineralization: principles and concepts in bioinorganic materials chemistry.* Oxford: Oxford University Press on Demand.

[B47] Marin F. Luquet G. (2008). *Unusually acidic proteins in biomineralization in Handbook of Biomineralization.* Germany: Wiley-VCH Verlag GmbH, 273–290. 10.1002/9783527619443.ch16

[B48] Marin F. Bundeleva I. Takeuchi T. Immel F. Medakovic D. (2016). Organic matrices in metazoan calcium carbonate skeletons: composition, functions, evolution. *J. Struct. Biol.* 196 98–106. 10.1016/j.jsb.2016.04.006 27085423

[B49] Mass T. Drake J. L. Haramaty L. Kim J. D. Zelzion E. Bhattacharya D. (2013). Cloning and characterization of four novel coral acid-rich proteins that precipitate carbonates *in vitro*. *Curr. Biol.* 23 1126–1131. 10.1016/j.cub.2013.05.007 23746634

[B50] Mass T. Putnam H. M. Drake J. L. Zelzion E. Gates R. D. Bhattacharya D. (2016). Temporal expression pattern of biomineralization proteins during early development in the stony coral *Pocillopora damicornis*. *Proc. R. Soc. B Biol. Sci.* 2016:283. 10.1098/rspb.2016.0322 PMC4855386 27122561

[B51] McLennan D. A. (2008). The concept of co-option: Why evolution often looks miraculous. *Evol. Educ. Outreach* 1 247–258. 10.1007/s12052-008-0053-8

[B52] Mello C. V. Lovell P. V. Wirthlin M. (2018). *Discovery of novel genes and other lineage-specific features through comparative genomics in Molecular-Genetic and Statistical Techniques for Behavioral and Neural Research.* 225–241. Amsterdam: Elsevier Inc., 10.1016/B978-0-12-804078-2.00010-6

[B53] Moberg F. Folke C. (1999). Ecological goods and services of coral reef ecosystems. *Ecol. Econ.* 29 215–233. 10.1016/S0921-8009(99)00009-9

[B54] Murdock D. J. E. (2020). The ‘biomineralization toolkit’ and the origin of animal skeletons. *Biol. Rev.* 2020:brv.12614. 10.1111/brv.12614 32447836

[B55] Murdock D. J. E. Donoghue P. C. J. (2011). Evolutionary origins of animal skeletal biomineralization. *Cells Tissues Organs* 194 98–102. 10.1159/000324245 21625061

[B56] Nguyen L.-T. Schmidt H. A. Von Haeseler A. Minh B. Q. (2015). IQ-TREE: a fast and effective stochastic algorithm for estimating maximum-likelihood phylogenies. *Mol. Biol. Evol.* 32 268–274. 25371430 10.1093/molbev/msu300 PMC4271533

[B57] Paps J. Holland P. W. H. (2018). Reconstruction of the ancestral metazoan genome reveals an increase in genomic novelty. *Nat. Commun.* 9 1–8. 29712911 10.1038/s41467-018-04136-5 PMC5928047

[B58] Peled Y. Drake J. L. Malik A. Almuly R. Lalzar M. Morgenstern D. (2020). Optimization of skeletal protein preparation for LC–MS/MS sequencing yields additional coral skeletal proteins in Stylophora pistillata. *BMC Mater.* 2:8 10.1186/s42833-020-00014-x PMC7115838 32724895

[B59] Ramos-Silva P. Marin F. (2015). Proteins as functional units of biocalcification – an overview. *Key Eng. Mater.* 672 183–190. 10.4028/www.scientific.net/KEM.672.183

[B60] Ramos-Silva P. Kaandorp J. Huisman L. Marie B. Zanella-Cléon I. Guichard N. (2013). The skeletal proteome of the coral *Acropora millepora*: the evolution of calcification by co-option and domain shuffling. *Mol. Biol. Evol.* 30 2099–2112. 10.1093/molbev/mst109 23765379 PMC3748352

[B61] Rastogi S. Liberles D. A. (2005). Subfunctionalization of duplicated genes as a transition state to neofunctionalization. *BMC Evol. Biol.* 5:28. 10.1186/1471-2148-5-28 15831095 PMC1112588

[B62] Richter D. J. Fozouni P. Eisen M. B. King N. (2018). Gene family innovation, conservation and loss on the animal stem lineage. *Elife* 7:e34226. 10.7554/eLife.34226 PMC6040629 29848444

[B63] Riehemann K. Sorg C. (1993). Sequence homologies between four cytoskeleton-associated proteins. *Trends Biochem. Sci.* 18 82–83. 10.1016/0968-0004(93)90159-K 8480366

[B64] Romano S. L. Palumbi S. R. (1996). Evolution of scleractinian corals inferred from molecular systematics. *Science* 271 640–642. 10.1126/science.271.5249.640

[B65] Schalchian-Tabrizi K. Minge M. A. Espelund M. Orr R. Ruden T. Jakobsen K. S. (2008). Multigene phylogeny of Choanozoa and the origin of animals. *PLoS One* 3:0002098. 10.1371/journal.pone.0002098 18461162 PMC2346548

[B66] Seppey M. Manni M. Zdobnov E. M. (2019). *BUSCO: Assessing genome assembly and annotation completeness in Methods in Molecular Biology.* Totowa: Humana Press Inc., 227–245. 10.1007/978-1-4939-9173-0_14 31020564

[B67] Singh T. R. Bansal A. (2019). *“Phylogenetic analysis: gene duplication and speciation,” in Encyclopedia of Bioinformatics and Computational Biology.* Amsterdam: Elsevier, 965–974. 10.1016/B978-0-12-809633-8.20176-3

[B68] Sinha S. Eisenhaber B. Lynn A. M. (2018). *“Predicting protein function using homology-based methods,” in Bioinformatics: Sequences, Structures, Phylogeny.* Berlin: Springer, 289–305.

[B69] Stolarski J. Kitahara M. V. Miller D. J. Cairns S. D. Mazur M. Meibom A. (2011). The ancient evolutionary origins of Scleractinia revealed by azooxanthellate corals. *BMC Evol. Biol.* 11:316. 10.1186/1471-2148-11-316 22034946 PMC3224782

[B70] Takeuchi T. Yamada L. Shinzato C. Sawada H. Satoh N. (2016). Stepwise evolution of coral biomineralization revealed with genome-wide proteomics and transcriptomics. *PLoS One* 11:0156424. 10.1371/journal.pone.0156424 27253604 PMC4890752

[B71] Tambutté S. Holcomb M. Ferrier-Pagès C. Reynaud S. Tambutté É Zoccola D. (2011). Coral biomineralization: from the gene to the environment. *J. Exp. Mar. Bio. Ecol.* 408 58–78. 10.1016/j.jembe.2011.07.026

[B72] Thongboonkerd V. Mcleish K. R. Arthur J. M. Klein J. B. (2002). Proteomic analysis of normal human urinary proteins isolated by acetone precipitation or ultracentrifugation. *Kidney Int.* 62:1461-1469. 10.1111/j.1523-1755.2002.kid565.x 12234320

[B73] True J. R. Carroll S. B. (2002). Gene co-option in physiological and morphological evolution. *Annu. Rev. Cell Dev. Biol.* 18 53–80. 10.1146/annurev.cellbio.18.020402.140619 12142278

[B74] Veron J. E. N. (2000). *Corals of the world.* Australia: Australian Institute of Marine Science.

[B75] Veron J. E. N. Hoegh-Guldberg O. Lenton T. M. Lough J. M. Obura D. O. Pearce-Kelly P. (2009). The coral reef crisis: the critical importance of <350 ppm CO_2_. *Mar. Pollut. Bull.* 58 1428–1436. 10.1016/J.MARPOLBUL.2009.09.009 19782832

[B76] Weiner S. (2003). An overview of biomineralization processes and the problem of the vital effect. *Rev. Mineral. Geochemistry* 54 1–29. 10.2113/0540001 28159795

[B77] WoRMS E. B. (2020). *World register of marine species (WoRMS).* Belgium: World register of marine species.

[B78] Ying H. Cooke I. Sprungala S. Wang W. Hayward D. C. Tang Y. (2018). Comparative genomics reveals the distinct evolutionary trajectories of the robust and complex coral lineages. *Genome Biol.* 19 1–24. 10.1186/s13059-018-1552-8 30384840 PMC6214176

[B79] Zoccola D. Innocenti A. Bertucci A. Tambutté Ć Supuran C. Tambutté S. (2016). Coral carbonic anhydrases: regulation by ocean acidification. *Mar. Drugs* 14:109. 10.3390/md14060109 27271641 PMC4926068

