# Supplementary material for: Evolution of Protein-Mediated Biomineralization in Scleractinian Corals
Source: Front Genet. 2021 Feb 2;12:618517. doi: 10.3389/fgene.2021.618517 (PMC7902050; doi:10.3389/fgene.2021.618517)
Supplement: Supplementary file 1 [file Data_Sheet_1.docx]

Supplementary Material

**Supplementary Table 1.** Species list used for the phylogenetic reconstruction analysis.

**Supplementary Table 2.** The list of all scleractinian SOM proteins used in this study, derived from the literature and their orthogroup assignment. Sequences assigned to the same orthogroup, yet they were not identified in the same ‘Scleractinia branch’, are marked with a different letter in the “branch ID” column. Sequences that were not assigned to any orthogroup are represented by “NAO”.

**Supplementary Table 3.** The list of duplication rates and SOM protein gain (proportional to the entire scleractinian SOM protein set) at the ancestral lineage and at the last duplication event.

**Supplementary Table 4.** Putative ortholog assignment of *Heliopora coerulea* transcripts to the known scleractinian SOM proteins.

**
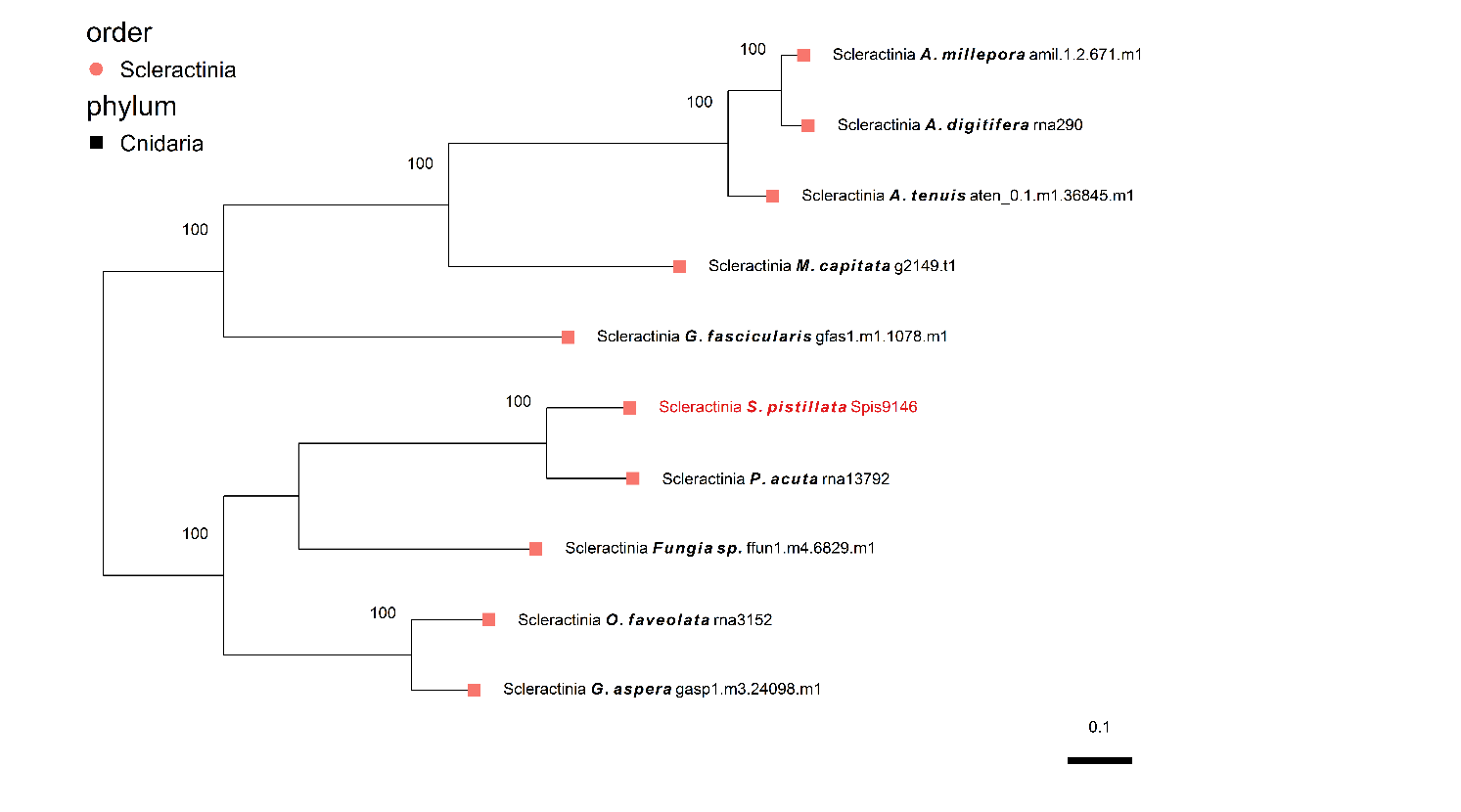
Supplementary Figure 1.** Rooted gene tree of the Coral Acid-Rich Protein 2 (CARP2, OG0012228) identified as the sole scleractinian-specific gene family composed of species from both the complex and robust scleractinian clades. Node points represent the phylum (shape) and order (color). Tips that are labeled red indicate the occurrence of scleractinian known SOM proteins. Node support values indicate percentage bootstrap values.

**
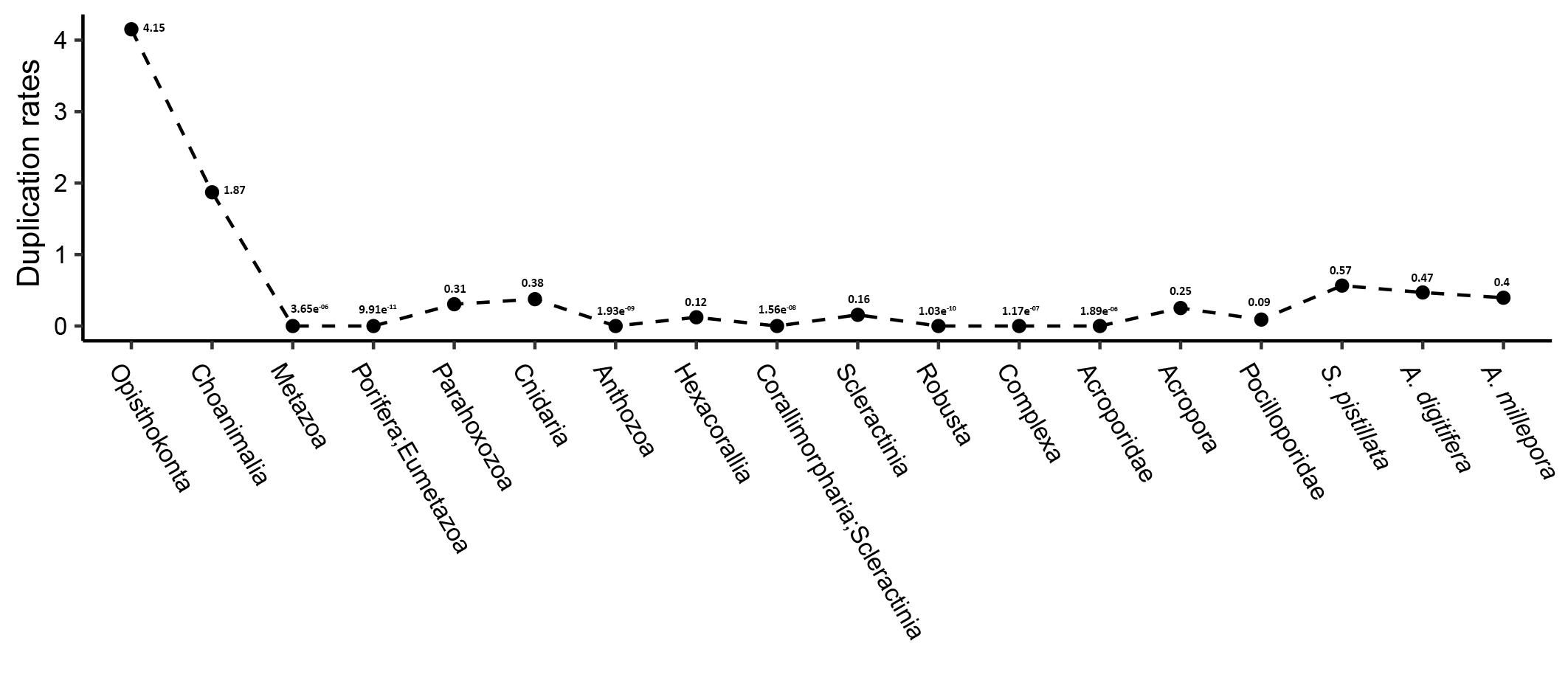
Supplementary Figure 2.** Duplication rates for each lineage leading to the known SOM proteins with species per node as inferred from Supplementary Table 3.

**
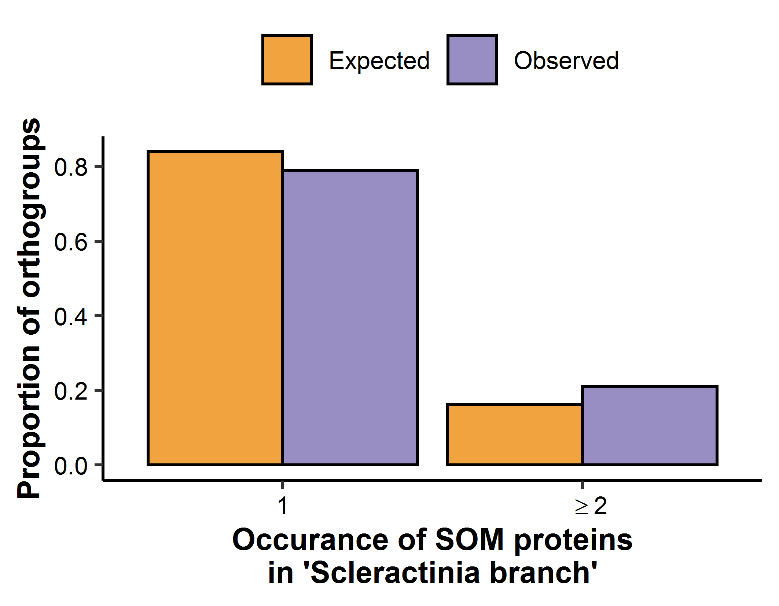
Supplementary Figure 3.** Distribution of SOM proteins found in ‘Scleractinia branches’ across all orthogroups found in our analysis (“Observed”) and the expected distribution, found through the permutation test (“Expected”).

**
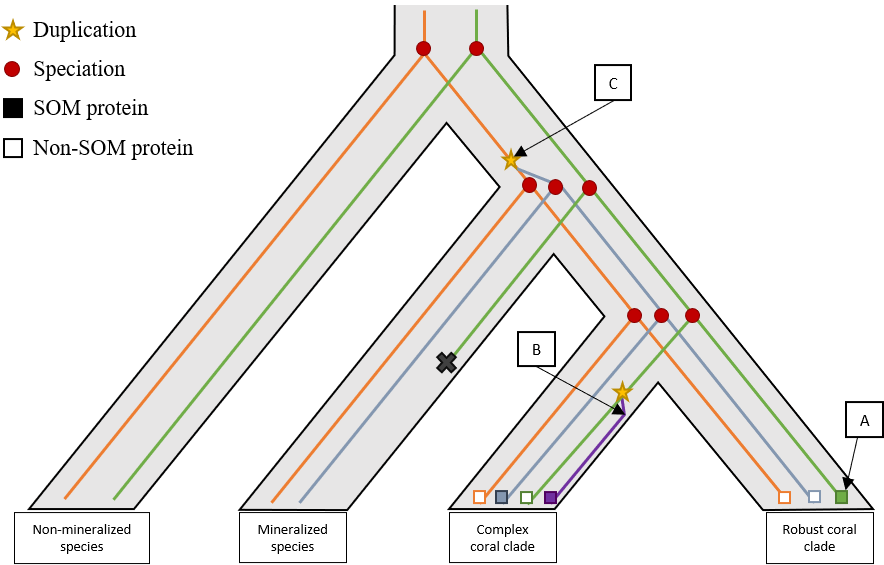
Supplementary Figure 4.** A gene trees/species tree simplifying the major evolutionary processes contributing to scleractinian SOM proteins evolution. The species tree is represented as light gray sleeves. Within the species tree two ancestral gene trees evolves and are represented in separate lines. In the root of the species tree, two ancestral genes are represented as green and orange lines. The genes are inherited to the sister groups by a speciation event (red circles) and might be lost in a particular lineage (black ‘X’). Within the gene tree, duplication events emerged at different points in the evolutionary time (yellows stars). Color filled squares represent sequences found in the SOM, while empty squares represent scleractinian sequences that were not identified in the SOM. (A) The independent co-option of a pre-existing molecular trait to serve a new role in the scleractinian skeleton formation. (B) A neofunctionalization process where a paralog sequence takes on a new function after a duplication event. (C) A subfunctionalization process where paralog sequences are carrying complementary functions after a duplication event.
